# Supplementary material for: Disentangling post-vaccination symptoms from early COVID-19
Source: eClinicalMedicine. 2021 Dec 1;42:101212. doi: 10.1016/j.eclinm.2021.101212 (PMC8635464; doi:10.1016/j.eclinm.2021.101212)
Supplement: Supplementary file 1 [file mmc1.pdf]

Supplementary Materials

Contents:

- Supplementary Methods
  - App development
  - Testing Kits
- Supplementary Results
  - Tables:
    - Supplementary Table S1. List of symptom questions asked by the COVID Symptom Study app.
    - Supplementary Table S2. List of local symptoms caused by the COVID Symptom Study app, caused by vaccination.
    - Supplementary Table S3: Properties and features of models used in this study.
    - Supplementary Table S4. Listing of clinical symptoms grouping.
    - Supplementary Table S5. Profiles of illness in symptomatic individuals after the 1st dose of vaccination (N=145) and 2nd dose (N=4).
    - Supplementary Table S6. Symptom prevalence and distribution during the first week after vaccination, in symptomatic individuals testing positive for SARS-CoV-2 infection.
    - Supplementary Table S7. Symptom prevalence and distribution during the first week after vaccination, in symptomatic individuals testing negative for SARS-CoV-2 infection (1:1 matched cohort).
    - Supplementary Table S8. Duration of individual symptoms after first vaccination (irrespective of symptom prevalence) in individuals testing positive or negative for SARS-CoV-2 (N=145 for each cohort).
    - Supplementary Table S9. Demographic information of vaccinated individuals testing positive (N=149) and a constructed cohort of equal size created by bootstrapping testing negative for SARS-CoV-2.
  - Figures:
    - Supplementary Figure S1. Profiles of illness in symptomatic individuals early post-vaccination, comparing symptom prevalence (symptom reported at any time during first week) in positive vs. negative cases (N=145 for each cohort), using bootstrapping to construct the negative cohort.
    - Supplementary Figure S2. Profiles of illness in symptomatic individuals early post-vaccination, comparing symptom prevalence (symptom reported at any time during first week) in individuals testing positive (N=145) vs. negative (N=12112) for SARS-CoV-2 infection.
    - Supplementary Figure S3. Correlation of symptoms experienced early post-vaccination in individuals testing positive (left image) or negative (right image) for SARS-CoV-2 infection (N=149).

Supplementary Methods

App development

In this prospective cohort study, data were acquired from CSS, through a mobile application for iPhone® and Android® users launched jointly by ZOE Limited. and KCL on 24 March 2020. [15] The COVID Symptoms Study app was tested before its launch, as well as before the release of any new version; software for repeatable and consistent data extraction, curation, and analytics was also engineered. [48]

The app is concurrently available to be download to Android and iOS, in both English and Swedish, noting the countries where it is currently available are the UK, US, and Sweden). The app was widely publicised in these countries during the peak of the pandemic, and new functionalities were added when appropriate to address the needs of the population, such as the registration of side-effects of the vaccination. Additionally, to keep the users engaged to the study, daily and weekly updates regarding research conducted using the data collected via the app are provided to participants (with notification through the app, email alerts, and posted on the website ZOE COVID Study (joinzoe.com)).

Testing Kits

SARS-CoV-2 testing kits were supplied by the Department of Health and Social Care, UK Government. These were sent to app users on their request.

Supplementary Results: Tables

Supplementary Table S 1. List of symptom questions asked by the COVID Symptom Study app. Questions were: Do you have (symptom)? Answers were yes/no, unless indicated otherwise.

**Symptom**	**COVID Symptom Study app question**
Fever	Fever (at least 37.8C or 100F)
Persistent Cough	Persistent cough (coughing a lot for more than an hour or 3 or more coughing episodes in 24 hours)
Fatigue	Unusual fatigue… (no; mild fatigue; severe fatigue/ I struggle to get out of bed)
Dyspnoea	Shortness of breath or trouble breathing (no; yes mild symptoms/ slight shortness of breath during ordinary activity: yes significant symptoms/ breathing is comfortable only at rest; yes, severe symptoms/ breathing is difficult even at rest).
Anosmia/Ageusia	Loss of smell / taste
Hoarse Voice	Unusually hoarse voice
Chest Pain	Unusual chest pain or tightness in your chest
Abdominal Pain	Unusual abdominal pain or stomach ache
Diarrhoea	Diarrhoea
Delirium	Confusion, disorientation or drowsiness
Eye Soreness (ophthalmalgia)	Do your eyes have any unusual eye-soreness or discomfort (e.g. light sensitivity, excessive tears, or pink/red eye)?
Loss of appetite (anorexia)	Skipping meals
Headache	Headache
Nausea	Nausea or vomiting
Dizziness	Dizziness or light-headedness
Sore Throat	Sore or painful throat
Myalgias	Unusual strong muscle pains or aches
Red Welts in Face and Lips	Raised, red, itchy welts on the skin or sudden swelling of the face or lips
Blisters on feet	Red/purple sores or blisters on your feet, including your toes
Sensitive Skin	Strange, unpleasant sensations in your skin like pins & needles or burning
Brain Fog	Loss of concentration or memory (brain fog)
Dysosmia/Dysgeusia	Altered smell / taste (things smell or taste different to usual)
Rhinorrhoea	Runny nose
Sneezing (sternutation)	Sneezing more than usual
Ear Pain (otalgia)	Earache

Lymphadenopathy	Swollen neck glands
Palpitations	Irregular heart beat

Supplementary Table S 2. List of local symptoms caused by the COVID Symptom Study app, caused by vaccination. Questions were: Do you have (symptom)? Answers were yes/no, unless indicated otherwise. These symptoms were excluded from this analysis.

**Symptom**	**COVID Symptom Study app question**
Tenderness	Tenderness near the site of injection
Pain	Arm pain
Redness	Redness near the site of injection
Swelling	Arm or local swelling
Warmth	Warmth near the site of injection
Bruising	Bruising near the site of injection
Swollen glands	Swollen glands in the armpit

Supplementary Table S 3. Properties and features of models used in this study. The input variables of each model (covariates) include both all the symptoms registered in the app (Supplementary Table S1), and/or the demographic information about the participant, including age, gender and BMI. The input variables also include either the patient received the first or second dose of the vaccine (defined as dose).

**Models**	**Input variables**	**Response variable**	**Model Characteristics**	**COVID-19 studies**
Bayesian mixed-effect model	Fixed effects: all symptoms (Supplementary Table S1).<br><br>Random effects: demographic information (age, BMI, gender), vaccine dose.	Test outcome – probabilistic outcome	Parametric model [49]	N/A
Logistic regression	All symptoms (Supplementary Table S1)demographic information (age, BMI, gender), and vaccine dose.	Test outcome – binary outcome	Parametric model	COVID-19 detection using self-reported symptoms [21]
Random forest	All symptoms (Supplementary Table S1)demographic information (age, BMI, gender), and vaccine dose.	Test outcome – binary outcome	Non-parametric model - intrinsic feature space transformation	Identification of early stage symptoms of SARS-Cov-2 infected patients [50]
Clinical symptom clustering	All symptoms (Supplementary Table S1).	Test outcome	Clinical association of symptoms.	N/A
NHS diagnostic criteria	Fever, Cough, Anosmia, Dysosmia	Test outcome	Screening criteria adopted by NHS	COVID-19 test referencing

Supplementary Table S 4. Listing of clinical symptoms grouping.

Clinical grouping	List of symptoms
Upper respiratory tract symptoms	anosmia, sore throat, rhinorrhoea, sneezing, ear pain, dysosmia
Respiratory	persistent cough, dyspnoea, chest pain, hoarse voice, plus any/all of the upper respiratory tract list
Systemic	fever, fatigue, delirium, headache, chills or shivers, eye soreness, myalgias, dizzy, light-headed, swollen glands [other than axillary lymph nodes in vaccinated arm], brain fog, irregular heartbeat
Abdominal	diarrhoea, abdominal pain, nausea, loss of appetite
Cutaneous	blisters on feet, sensitive skin and red welts on face and lips

Supplementary Table S 5. Profiles of illness in symptomatic individuals after the 1st dose of vaccination (N=145) and 2nd dose (N=4). Cells show absolute number (percentage in parentheses) of symptomatic individuals for symptoms reported at any time during first week, in positive vs. negative cases**.**

	**First dose (N=145)**		**Second dose (N=4)**	
**Symptoms**	**Positive test for SARS-CoV-2 infection**	**Negative test for SARS-CoV-2 infection**	**Positive test for SARS-CoV-2 infection**	**Negative test for SARS-CoV-2 test infection**
Headache	86 (59.3)	101 (70.0)	3 (0.75)	3 (0.75)
Chills or Shivers	56 (38.6)	60 (41.3)	0 (0.0)	1(0.25)
Fever	40 (27.6)	36 (24.8)	1(0.25)	0 (0.0)
Sore Throat	32 (22.1)	117 (11.7)	1(0.25)	0 (0.0)
Myalgias	29 (20.0)	25 (17.2)	2 (0.50)	0 (0.0)
Rhinorrhoea	27 (18.6)	19 (13.1)	2 (0.50)	1(0.25)
Sneezing	23 (15.9)	12 (8.3)	1(0.25)	0 (0.0)
Nausea	22 (15.2)	26 (17.9)	0 (0.0)	0 (0.0)
Dizziness	22 (15.2)	31 (21.4)	0 (0.0)	1(0.25)
Loss of Appetite	19 (13.1)	17 (11.7)	0 (0.0)	0 (0.0)
Abdominal Pain	16 (11.0)	18 (12.4)	0 (0.0)	2 (0.50)
Diarrhoea	14 (9.7)	10 (6.9)	0 (0.0)	0 (0.0)
Eye Soreness	12 (8.3)	13 (9.0)	0 (0.0)	0 (0.0)

Persistent Cough	12 (8.3)	4 (2.8)	0 (0.0)	0 (0.0)
Hoarse Voice	12 (8.3)	5 (3.4)	2 (0.50)	0 (0.0)
Chest Pain	11 (7.6)	7 (4.8)	0 (0.0)	0 (0.0)
Delirium	10 (6.9)	5 (3.4)	0 (0.0)	0 (0.0)
Fatigue	9 (6.2)	9 (6.2)	0 (0.0)	1(0.25)
Brain Fog	9 (6.2)	13 (9.0)	0 (0.0)	0 (0.0)
Dysosmia	7 (4.8)	6 (4.1)	1(0.25)	0 (0.0)
Sensitive Skin	6 (4.1)	4 (2.8)	0 (0.0)	0 (0.0)
Anosmia	5 (3.4)	2 (1.4)	0 (0.0)	0 (0.0)
Ear Pain	4 (2.8)	6 (4.1)	0 (0.0)	0 (0.0)
Swollen Glands	4 (2.8)	6 (4.1)	1(0.25)	0 (0.0)
Palpitations	3 (2.1)	11 (7.6)	0 (0.0)	0 (0.0)
Red Welts FL	1(0.7)	1(0.7)	0 (0.0)	0 (0.0)
Blisters on Feet	1(0.7)	0 (0.0)	0 (0.0)	0 (0.0)
Dyspnoea	0 (0.0)	0 (0.0)	0 (0.0)	0 (0.0)

Supplementary Table S 6. Symptom prevalence and distribution during the first week after vaccination, in symptomatic individuals testing positive for SARS-CoV-2 infection. Data report number (percentage in parentheses) of individuals with each symptom on each day after first vaccination (N = 145).

	**Positive SARS-CoV-2**							
**Days after vaccination**	0	1	2	3	4	5	6	7
Headache	1 (0.7)	10 (6.9)	11 (7.6)	29 (20.0)	15 (10.3)	12 (8.3)	23 (15.9)	31 (21.4)
Chills or Shivers	0 (0.0)	6 (4.1)	7 (4.8)	15 (10.3)	10 (6.9)	10 (6.9)	12 (8.3)	12 (8.3)
Myalgias	1 (0.7)	1 (0.7)	2 (1.4)	10 (6.9)	2 (1.4)	3 (2.1)	8 (5.5)	15 (10.3)
Fever	0 (0.0)	8 (5.5)	6 (4.1)	8 (5.5)	2 (1.4)	5 (3.4)	8 (5.5)	8 (5.5)
Dizziness	0 (0.0)	2 (1.4)	2 (1.4)	7 (4.8)	6 (4.1)	3 (2.1)	5 (3.4)	8 (5.5)
Sneezing	0 (0.0)	1 (0.7)	1 (0.7)	6 (4.1)	2 (1.4)	4 (2.8)	2 (1.4)	12 (8.3)
Nausea	0 (0.0)	3 (2.1)	2 (1.4)	6 (4.1)	2 (1.4)	1 (0.7)	3 (2.1)	7 (4.8)
Sensitive Skin	0 (0.0)	0 (0.0)	0 (0.0)	6 (4.1)	0 (0.0)	0 (0.0)	3 (2.1)	0 (0.0)
Rhinorrhoea	0 (0.0)	3 (2.1)	1 (0.7)	4 (2.8)	6 (4.1)	3 (2.1)	7 (4.8)	11 (7.6)
Eye Soreness	0 (0.0)	1 (0.7)	0 (0.0)	4 (2.8)	2 (1.4)	0 (0.0)	4 (2.8)	7 (4.8)
Loss of Appetite	0 (0.0)	4 (2.8)	2 (1.4)	3 (2.1)	0 (0.0)	4 (2.8)	7 (4.8)	8 (5.5)
Chest Pain	0 (0.0)	0 (0.0)	0 (0.0)	3 (2.1)	0 (0.0)	1 (0.7)	10 (6.9)	3 (2.1)
Sore Throat	1 (0.7)	3 (2.1)	5 (3.4)	3 (2.1)	2 (1.4)	2 (1.4)	14 (9.7)	14 (9.7)
Brain Fog	0 (0.0)	0 (0.0)	0 (0.0)	3 (2.1)	1 (0.7)	2 (1.4)	7 (4.8)	7 (4.8)
Hoarse Voice	0 (0.0)	1 (0.7)	0 (0.0)	2 (1.4)	0 (0.0)	1 (0.7)	5 (3.4)	9 (6.2)
Anosmia	0 (0.0)	0 (0.0)	0 (0.0)	2 (1.4)	2 (1.4)	0 (0.0)	1 (0.7)	1 (0.7)
Abdominal Pain	0 (0.0)	2 (1.4)	1 (0.7)	2 (1.4)	1 (0.7)	6 (4.1)	4 (2.8)	6 (4.1)
Diarrhoea	0 (0.0)	1 (0.7)	2 (1.4)	2 (1.4)	2 (1.4)	2 (1.4)	0 (0.0)	9 (6.2)
Dysosmia	0 (0.0)	1 (0.7)	0 (0.0)	2 (1.4)	1 (0.7)	0 (0.0)	7 (4.8)	5 (3.4)
Ear Pain	0 (0.0)	0 (0.0)	0 (0.0)	2 (1.4)	1 (0.7)	0 (0.0)	0 (0.0)	3 (2.1)
Red Welts FL	0 (0.0)	0 (0.0)	0 (0.0)	1 (0.7)	0 (0.0)	0 (0.0)	0 (0.0)	0 (0.0)
Fatigue	0 (0.0)	1 (0.7)	0 (0.0)	1 (0.7)	2 (1.4)	0 (0.0)	4 (2.8)	6 (4.1)
Persistent Cough	0 (0.0)	0 (0.0)	4 (2.8)	0 (0.0)	1 (0.7)	4 (2.8)	7 (4.8)	6 (4.1)
Swollen Glands	0 (0.0)	0 (0.0)	0 (0.0)	0 (0.0)	1 (0.7)	0 (0.0)	4 (2.8)	2 (1.4)
Blisters on Feet	0 (0.0)	0 (0.0)	0 (0.0)	0 (0.0)	0 (0.0)	0 (0.0)	4 (2.8)	0 (0.0)
Dyspnoea	0 (0.0)	0 (0.0)	0 (0.0)	0 (0.0)	0 (0.0)	0 (0.0)	0 (0.0)	0 (0.0)

Supplementary Table S 7. Symptom prevalence and distribution during the first week after vaccination, in symptomatic individuals testing negative for SARS-CoV-2 infection (1:1 matched cohort). Data report number (percentage in parentheses) of individuals with each symptom on each day after first vaccination (N=145).

	**Negative SARS-CoV-2**							
**Days after vaccination**	0 (0.0)	1 (0.7)	2	3	4	5	6	7
Headache	1 (0.7)	13 (9.0)	17 (11.7)	16 (11.0)	30 (20.7)	23 (15.9)	17 (11.7)	26 (17.9)
Chills or Shivers	0 (0.0)	11 (7.6)	10 (6.9)	8 (5.5)	17 (11.7)	7 (4.8)	5 (3.4)	14 (9.7)
Myalgias	0 (0.0)	2 (1.4)	5 (3.4)	1 (0.7)	6 (4.1)	8 (5.5)	5 (3.4)	7 (4.8)
Fever	0 (0.0)	6 (4.1)	5 (3.4)	6 (4.1)	8 (5.5)	7 (4.8)	7 (4.8)	6 (4.1)
Dizziness	0 (0.0)	4 (2.8)	2 (1.4)	11 (7.6)	10 (6.9)	8 (5.5)	7 (4.8)	5 (3.4)
Sneezing	0 (0.0)	1 (0.7)	0 (0.0)	4 (2.8)	4 (2.8)	1 (0.7)	1 (0.7)	4 (2.8)
Nausea	1 (0.7)	2 (1.4)	3 (2.1)	4 (2.8)	11 (7.6)	3 (2.1)	3 (2.1)	13 (9.0)
Sensitive Skin	0 (0.0)	0 (0.0)	0 (0.0)	1 (0.7)	1 (0.7)	0 (0.0)	0 (0.0)	2 (1.4)
Rhinorrhoea	0 (0.0)	0 (0.0)	1 (0.7)	2 (1.4)	5 (3.4)	11 (7.6)	2 (1.4)	6 (4.1)
Eye Soreness	0 (0.0)	2 (1.4)	2 (1.4)	4 (2.8)	1 (0.7)	5 (3.4)	3 (2.1)	0 (0.0)
Loss of Appetite	0 (0.0)	0 (0.0)	5 (3.4)	2 (1.4)	4 (2.8)	3 (2.1)	2 (1.4)	2 (1.4)
Chest Pain	0 (0.0)	0 (0.0)	1 (0.7)	1 (0.7)	1 (0.7)	2 (1.4)	2 (1.4)	1 (0.7)
Sore Throat	0 (0.0)	1 (0.7)	1 (0.7)	2 (1.4)	4 (2.8)	9 (6.2)	2 (1.4)	3 (2.1)
Brain Fog	0 (0.0)	0 (0.0)	5 (3.4)	4 (2.8)	1 (0.7)	1 (0.7)	6 (4.1)	3 (2.1)
Hoarse Voice	0 (0.0)	0 (0.0)	1 (0.7)	0 (0.0)	2 (1.4)	0 (0.0)	1 (0.7)	2 (1.4)
Anosmia	0 (0.0)	0 (0.0)	0 (0.0)	1 (0.7)	1 (0.7)	0 (0.0)	0 (0.0)	0 (0.0)
Abdominal Pain	0 (0.0)	1 (0.7)	1 (0.7)	3 (2.1)	4 (2.8)	4 (2.8)	7 (4.8)	4 (2.8)
Diarrhoea	0 (0.0)	0 (0.0)	2 (1.4)	0 (0.0)	3 (2.1)	5 (3.4)	1 (0.7)	3 (2.1)
Dysosmia	0 (0.0)	1 (0.7)	2 (1.4)	0 (0.0)	1 (0.7)	4 (2.8)	0 (0.0)	4 (2.8)
Ear Pain	0 (0.0)	1 (0.7)	0 (0.0)	1 (0.7)	2 (1.4)	0 (0.0)	1 (0.7)	3 (2.1)
Red Welts FL	0 (0.0)	0 (0.0)	0 (0.0)	0 (0.0)	0 (0.0)	0 (0.0)	0 (0.0)	1 (0.7)
Fatigue	0 (0.0)	2 (1.4)	4 (2.8)	2 (1.4)	0 (0.0)	0 (0.0)	1 (0.7)	1 (0.7)
Persistent Cough	0 (0.0)	0 (0.0)	0 (0.0)	0 (0.0)	2 (1.4)	3 (2.1)	0 (0.0)	0 (0.0)
Swollen Glands	0 (0.0)	2 (1.4)	1 (0.7)	1 (0.7)	2 (1.4)	0 (0.0)	1 (0.7)	0 (0.0)
Blisters on Feet	0 (0.0)	0 (0.0)	0 (0.0)	0 (0.0)	0 (0.0)	0 (0.0)	0 (0.0)	0 (0.0)
Dyspnoea	0 (0.0)	0 (0.0)	0 (0.0)	0 (0.0)	0 (0.0)	0 (0.0)	0 (0.0)	0 (0.0)

Supplementary Table S 8. Duration of individual symptoms after first vaccination (irrespective of symptom prevalence) in individuals testing positive or negative for SARS-CoV-2 (N=145 for each cohort). Median value of symptom duration ([0.25; 0.75] quantiles) is detailed per symptom. Differences in duration distribution per symptom were assessed using Mann-Whitney-U tests. P-value refers to the result of the statistical test.

Symptoms	Positive SARS-CoV-2	Negative SARS-CoV-2	P-Value
Headache	1 [1; 2]	1 [1; 2]	0.4428
Chills or Shivers	1 [1; 1]	1 [1; 1]	0.7550
Myalgias	1 [1; 2]	1 [1; 1]	0.4489
Fever	1 [1; 1]	1 [1; 1]	0.1599
Dizziness	1 [1; 2]	1 [1; 2]	0.7303
Sneezing	1 [1; 1]	1 [1; 1.25]	0.4415
Nausea	1 [1; 1]	1 [1; 1.75]	0.1033
Sensitive Skin	1 [1; 1.75]	1 [1; 1]	0.6667
Rhinorrhoea	1 [1; 1]	1 [1; 1]	0.9383
Eye Soreness	1 [1; 2]	1 [1; 1]	0.3634
Loss of Appetite	1 [1; 1.5]	1 [1; 1]	0.1015
Chest Pain	1 [1; 2]	1 [1; 1]	0.4314
Sore Throat	1 [1; 2]	1 [1; 1]	0.1747
Brain Fog	2 [1; 3]	1 [1; 2]	0.2129
Hoarse Voice	1 [1; 1.25]	1 [1; 1]	1.0000
Anosmia	1 [1; 1]	1 [1; 1]	1.0000
Abdominal Pain	1 [1; 1.25]	1 [1; 1]	0.8707
Diarrhoea	1 [1; 1]	1 [1; 2]	0.0896
Dysosmia	1 [1; 3.5]	2 [1; 2]	0.9825
Ear Pain	1 [1; 1.5]	1 [1; 1.75]	1.0000
Red Welts FL	1 [1; 1]	1 [1; 1]	1.0000
Fatigue	1 [1; 1]	1 [1; 1]	0.7353
Persistent Cough	1.5 [1; 2.25]	1 [1; 1.25]	0.5077
Swollen Glands	1 [1; 1.75]	1 [1; 1]	0.8000
Blisters on Feet	4 [4; 4]	N/A	N/A
Dyspnoea	N/A	N/A	N/A
Delirium	1 [1; 1.75]	2 [1; 3]	0.4775
Palpitations	1 [1; 1]	1 [1; 1.5]	0.9066

Supplementary Table S 9. Demographic information of vaccinated individuals testing positive (N=149) and a constructed cohort of equal size created by bootstrapping testing negative for SARS-CoV-2. Demographic information for the negative SARS-CoV-2 cohort was estimated based on the selected bootstrapped samples: thus, for most values the range is presented (as [Minimum; Maximum]); when appropriate median value [IQR] is presented (age, BMI). BMI: Body Mass Index. IQR: Inter Quartile Range.

	**Vaccinated Cohort**					
	**Positive test for SARS-CoV-2 infection**			**Negative test for SARS-CoV-2 infection**		
	**O-AZ**	**PB**	**Full cohort**	**O-AZ**	**PB**	**Full cohort**
Number	72	77	149	72	77	149
Males (%)	37.0	27.6	32.2	[36.9; 37.0]	[27.6; 27.6]	[32.2; 32.2]
Age, years (median [IQR])	62.0 [50.0; 71.0]	59.0 [50.0; 67.5]	61.0 [50.0; 70.0]	62.1 [49.3; 71.0]	59.4 [50.5; 67.8]	60.7 [50.3; 70.4]
BMI (median [IQR])	25.0 [22.7; 28.0]	26.1 [23.5; 29.3]	25.4 [23.4; 29.2]	25.1 [22.9; 28.3]	26.1 [23.6; 29.5]	25.6 [23.2; 29.0]
Lung disease (%)	11.1	11.8	11.4	[5.5; 15.1]	[5.3; 22.4]	[7.4; 14.1]
Kidney Disease (%)	0.0	1.3	0.7	[0.0; 4.1]	[0.0; 5.3]	[0.0; 3.4]
Diabetes (%)	5.5	6.6	6.0	[2.7; 10.9]	[2.6; 7.9]	[2.7; 8.1]
Heart Disease (%)	9.7	5.3	7.4	[2.7; 10.9]	[5.3; 11.8]	[4.0; 7.4]
Cancer (%)	0.0	5.3	2.7	[0.0; 6.9]	[0.0; 3.9]	[0.7; 4.7]
Healthcare workers (%)	0.0	13.2	6.7	[0.0; 2.7]	[6.6; 14.5]	[0.7; 5.4]
Visit to hospital (%)	1.4	1.3	1.3	[0.0; 1.4]	[0.0; 3.9]	[0.0; 2.0]

Supplementary Results: Figures

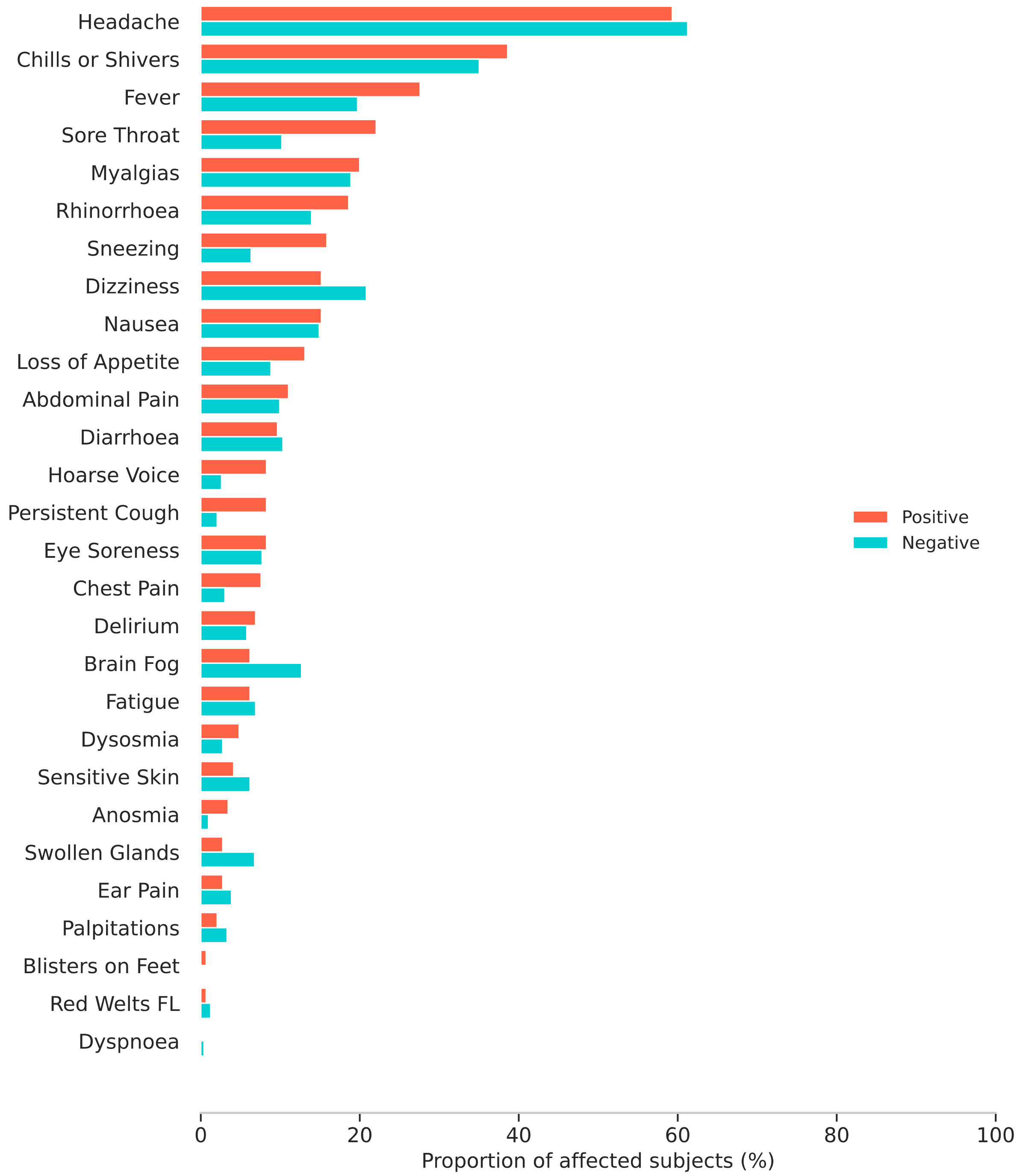

Supplementary Figure S 1. Profiles of illness in symptomatic individuals early post-vaccination, comparing symptom prevalence (symptom reported at any time during first week) in positive vs. negative cases (N=145 for each cohort), using bootstrapping to construct the negative cohort. The confidence interval (black bar) for the negative population is due to use of bootstrapping to create the negative population.

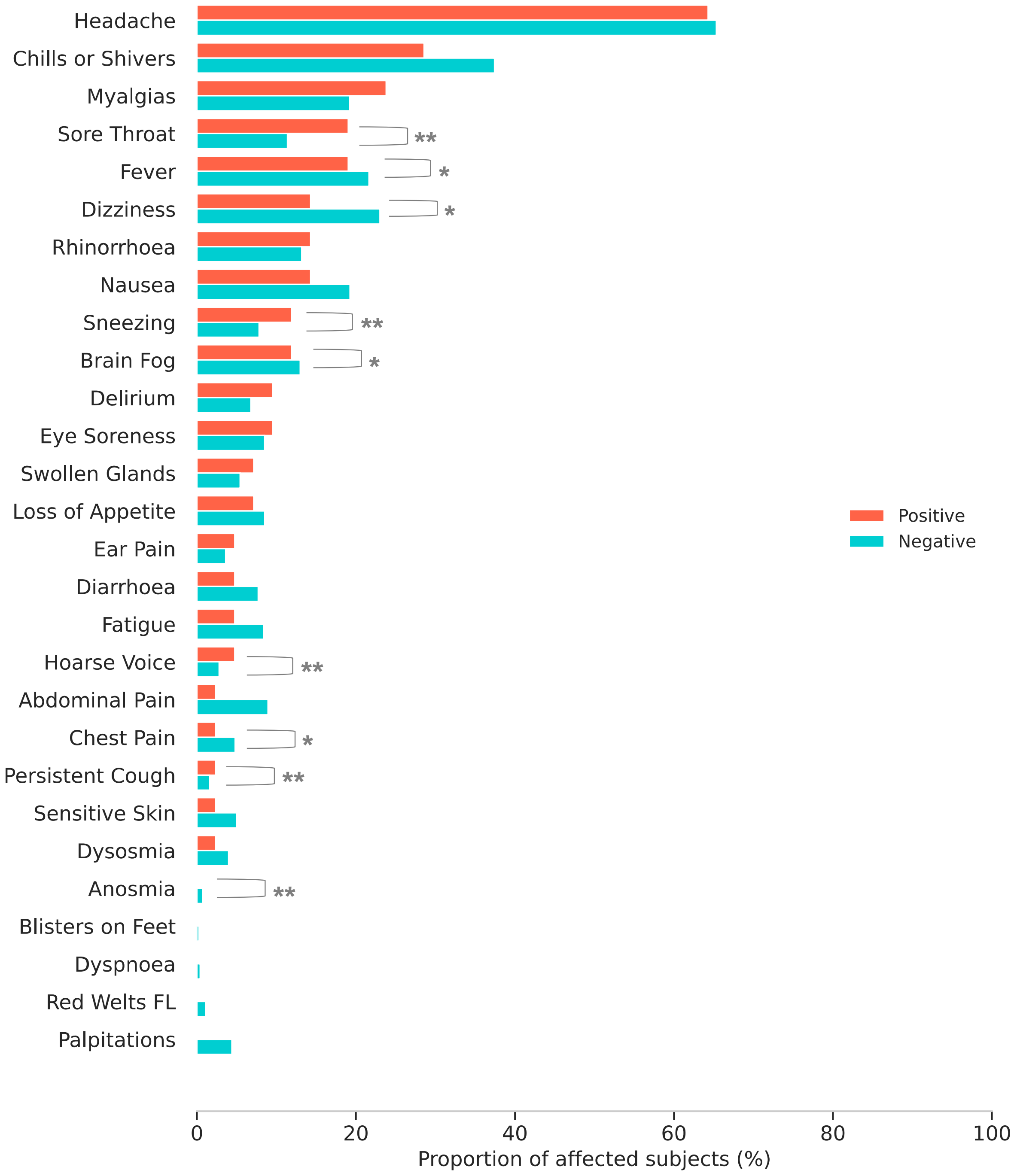

Supplementary Figure S 2. Profiles of illness in symptomatic individuals early post-vaccination, comparing symptom prevalence (symptom reported at any time during first week) in individuals testing positive (N=145) vs. negative (N=12112) for SARS-CoV-2 infection. *p < 0.05 **p < 0.01.

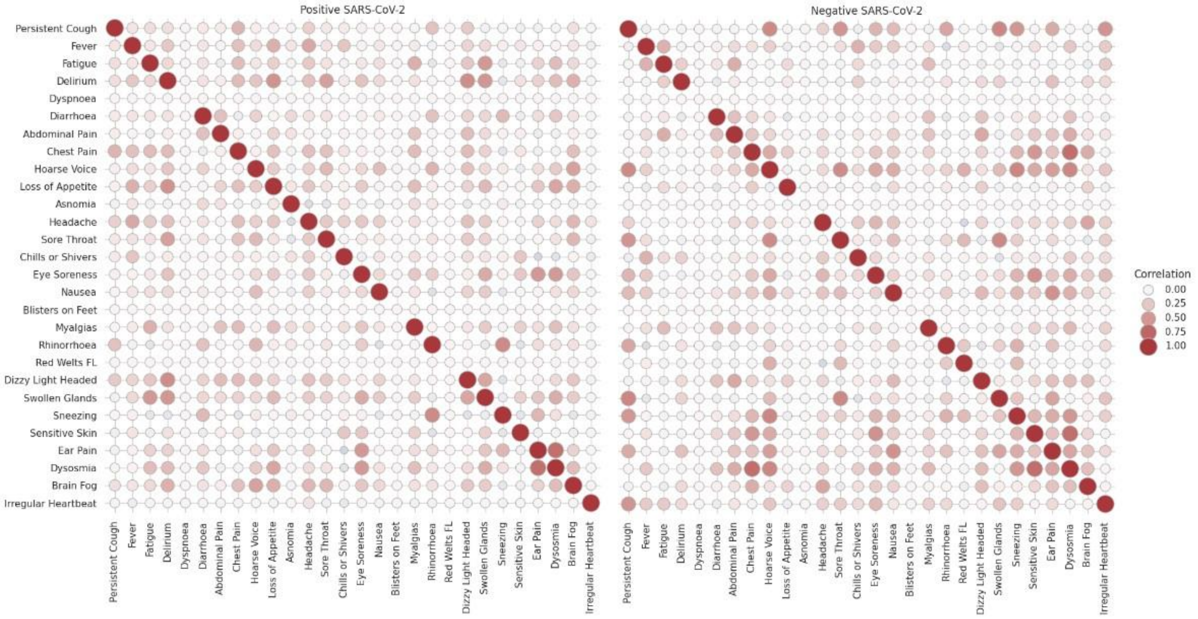

Supplementary Figure S 3. Correlation of symptoms experienced early post-vaccination in individuals testing positive (left image) or negative (right image) for SARS-CoV-2 infection (N=149). The colour and size of the marker encode the Spearman-rank correlation. Darker colours represent higher correlation of symptoms.
